# Determining the anatomical site in knee radiographs using deep learning

**DOI:** 10.1038/s41598-022-08020-7

**Published:** 2022-03-07

**Authors:** Anton S. Quinsten, Lale Umutlu, Michael Forsting, Kai Nassenstein, Aydin Demircioğlu

**Affiliations:** Institute of Diagnostic and Interventional Radiology and Neuroradiology, University Hospital Essen, Hufelandstr. 55, 45147 Essen, Germany

**Keywords:** Machine learning, Bone, Bone imaging, Radiography

## Abstract

An important quality criterion for radiographs is the correct anatomical side marking. A deep neural network is evaluated to predict the correct anatomical side in radiographs of the knee acquired in anterior–posterior direction. In this retrospective study, a ResNet-34 network was trained on 2892 radiographs from 2540 patients to predict the anatomical side of knees in radiographs. The network was evaluated in an internal validation cohort of 932 radiographs of 816 patients and in an external validation cohort of 490 radiographs from 462 patients. The network showed an accuracy of 99.8% and 99.9% on the internal and external validation cohort, respectively, which is comparable to the accuracy of radiographers. Anatomical side in radiographs of the knee in anterior–posterior direction can be deduced from radiographs with high accuracy using deep learning.

## Introduction

Conventional radiography is still one of the most often used imaging modalities, with an estimated 275 million exams performed in the U.S. in 2016.

Since the anatomical side cannot be reliable identified on radiograph due to symmetrical human anatomy or possible anatomical variants such as a situs inversus, it is necessary that an anatomical side marker (ASM) is given radiographs to allow subsequent reliable identification of the anatomical side^1^. Missing ASM in radiographs may have a serious impact on patients’ safety, since an incorrect identification of the anatomical side can have profound effects on the patient’s treatment and, in worst case, can cause patient’s death. For example, it has been reported, that a thoracostomy was performed on the wrong side in a premature baby with pneumothorax due to lack of an ASM in the radiograph resulting in children’s death^2^. Unfortunately, this is not an isolated case, and many case reports can be found in which a missing ASM in radiographs has led to malpractice^3–6^. Despite the great clinical importance and the legal requirement for side marking in radiograph images incorrect or missing ASM frequently occur in clinical routine^7–10^. In a study by Titley et al. correct ASM placement were only observed in 32%^11^.

In a study by Tugwell et al., 92% of the radio-opaque anatomical side markers, which were placed in the primary collimation field outside the anatomical area, were contaminated with various organisms including *Staphylococcus* and *Bacillus* species^12^. Thus, if the prescribed hygiene measures are not adhered to, the ASM can act therefore as a superspreader. Since hygienically cleaning the ASM after each use significantly affects the workflow, digital side markers (DSM) could therefore act as an alternative^13^. While DSM were already in common use, they were only considered recently during the COVID-19 pandemic as good practice. Unfortunately, placing a DSM is equally well prone to error or might be forgotten^8^, the placement is post-exposure and marker corrections can be performed more easily without repeating the acquisition^14^. An automation of the placement of DSMs could therefore be desirable.

A key obstruction to the automation of the placement of DSM is the huge variability in the scans, as radiographs are taken for multiple reasons in different patients. There are special quality requirements for newborns, infants, children and adolescents as each will in general have very different imaging conditions. In addition, fractures, implants, screws and other stabilization artifacts as well as tubes can also obstruct the radiograph. This huge variability makes classical image processing approaches very complex and therefore error prone.

Deep learning techniques on the other hand have been shown to be able to deal with large variability in images^15^. Therefore, a deep neural network could be used to predict the anatomical side of radiographs to which in turn can be used to place the correct DSM onto radiographs. In this study, it is demonstrated that a deep neural network can predict the correct anatomical side of radiographs of the knee acquired in anterior–posterior view.

## Materials and methods

### Ethical statement

The study was approved by the institutional ethics board (Ethics Committee of the Medical Faculty of the University Duisburg-Essen, Approval Number: 21-10063-BO). Written informed consent was waived by the ethics board due to the retrospective nature of the study. All methods and procedures were performed in accordance with the relevant guidelines and regulations.

### Patient cohorts

Training and internal validation cohorts were gathered by querying the in-house radiological information system (University Hospital Essen). The training cohort was randomly selected from all patients who had a radiograph of the knee in anterior–posterior direction acquired between 1.1.2009 and 31.12.2018. From each year, 300 radiographs were included into the training cohort. Radiographs were excluded for the following reasons: (a) not AP view (b) both knees were acquired (c) knee not fully visible (knee was cut or another body part was acquired) (d) low image quality (e) fibula missing.

The internal validation cohort was gathered in the same manner by including 1000 randomly selected radiographs of the knee acquired between 1.1.2019 and 31.12.2020. The same procedure, inclusion criteria as well as exclusion criteria were applied as for the training cohort. In addition, to avoid positive bias, radiographs of patients that were already included in the training cohort were removed from the validation cohort.

A second, external validation cohort was collected from a neighboring hospital (Elisabeth Krankenhaus Essen). Using their radiological information system, all patients with a knee radiograph between 1.1.2021 and 31.5.2021 were gathered.

**Figure 1:**
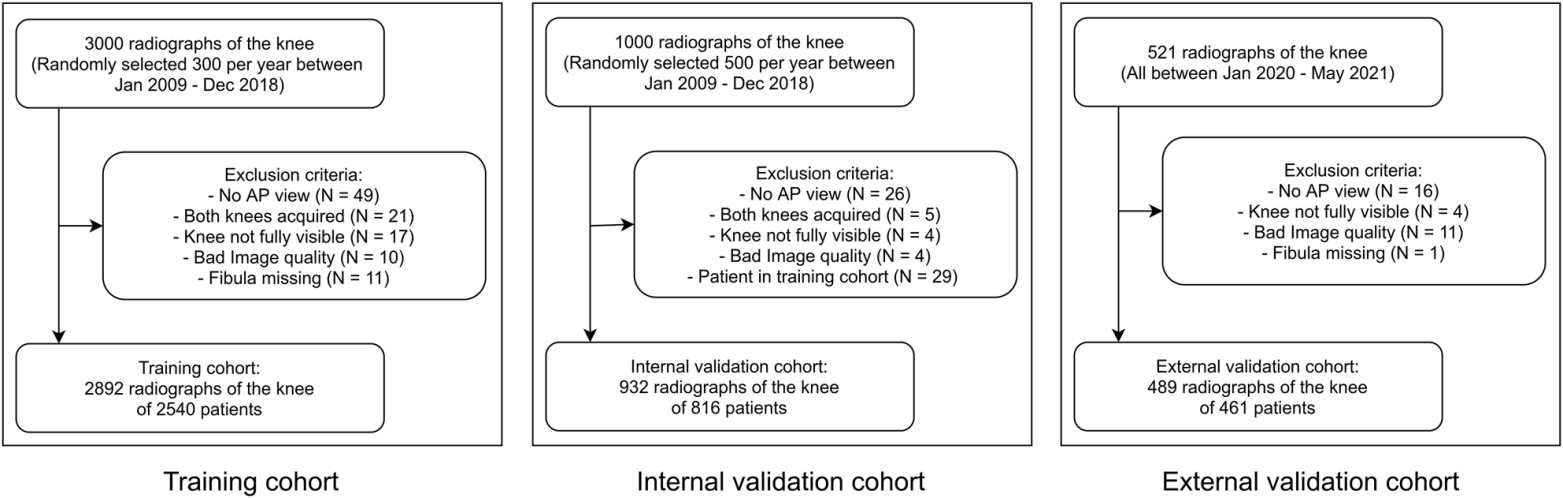
Patient flowcharts for all cohorts with inclusion and exclusion criteria.

Altogether, the training set consisted of 2892 radiographs from 2540 patients, the internal validation cohort of 932 radiographs from 816 patients and the external validation cohort of 490 radiographs from 462 patients (Fig. 1).

### Scanners and acquisition parameters

Radiographs in the training and validation cohort were acquired mainly on scanners from Siemens (Siemens Healthineers, Erlangen, Germany) and AGFA (AGFA Healthcare, Mortsel, Belgium). The external validation cohort was acquired using Canon (Canon Europe, London, UK) scanners (Table 1). On average, the radiographs were acquired with 64.8 kVp (range 54.9–75.0), 64.5 kVp (range 54.9–76.8), and 7.7 mAs (range 1–62) and 7.5 mAs (range 1–35) in the training and internal validation cohort respectively. For the external validation cohort these parameters were not available in the DICOM tags.

**Table 1:** Overview of the scanners used for the acquisition of the radiographs.

	All (N = 4314)	Train (N = 2892)	Internal Validation (N = 932)	External Validation (N = 489)
SIEMENS (Multix Top, Vertix 3D, Ysio, Ysio Max)	2458	1557	901	0
AGFA (CR 58, Solo, 51xx, Compact Plus)	1305	1304	1	0
CANON (CXDI)	466	0	0	465
Other	85	31	30	24

Scanners with less than 50 examinations were gathered into the “Other” group.

### Removing anatomical side markers

**Figure 2:**
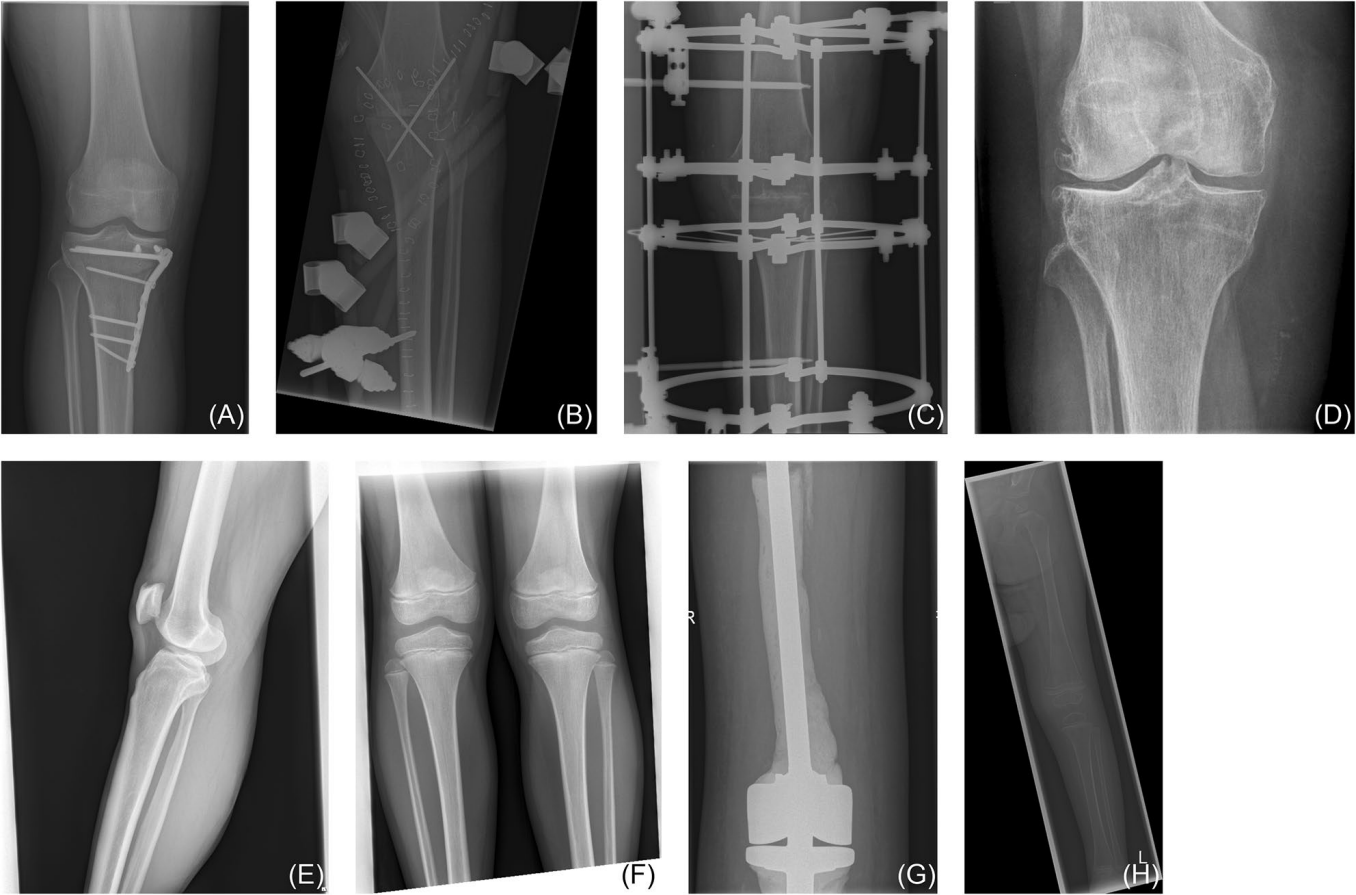
Knee radiographs for nine patients. The upper row depicts radiographs that were included into the study (images were cropped to remove the anatomic side markers) while the lower row shows examples of radiographs that were excluded. (**A**) Male patient (40.1 years) (**B**) Male patient (34.4 years) (**C**) Male patient (34.5 years) (**D**) Female patient (76.2 years) (**E**) Male patient (61.2 years), excluded because of lateral view (**F**) Male patient (11.1 years) excluded because of both knee are visible (**G**) Female patient (73.2 years) excluded because of knee is not fully visible (**H**) Female patient (1.9 years) excluded because of low image quality.

As placing an anatomical side marker is a major quality criterion, it can be expected that nearly all the data will contain an ASM. Without any processing, a network could “cheat” during training and use the ASM to determine the anatomical side instead of using the x-ray of the knee. The network would then fail completely on data without ASMs. Therefore, all ASMs need to be removed for training. Care needs to be taken, as the removal could introduce an unexpected bias into the model: In clinical routine, right ASMs are prominently placed on the right side (corresponding to the left side of the image), while left ASMs are placed on the left side. Therefore, if the ASMs are simply removed, e.g., by black rectangles, this would give away strong evidence to the anatomical side, and the network could determine it simply by observing on which side the visible modification has taken place. Unfortunately, removing the ASMs without any visible evidence is not easy, as these are sometimes placed very close to or even inside the knee tissue. Therefore, the ASMs were first marked in all radiographs in the training cohort and subsequently removed by cropping the radiographs to an area where the ASMs are not visible (Fig. 2). For the validation cohorts this procedure is not necessary, as the network never saw these markers and did not learn their meaning so cannot use them for inference.

### Annotations

Anatomical labeling was determined by reading the corresponding DICOM tags. Nonetheless, as the tags might contain errors, all images were checked by the radiographer (20 years of experience) and corrected where necessary.

### Preprocessing

After cropping, the cropped radiographs were checked a second time to make sure no kind of side markers were still present in the data. All radiographs were then reduced from 12-bit to 8-bit by simple linear rescaling and then rescaled to a size of 256 × 256.

### Neural network

For modeling, a standard network, the ResNet-34, pretrained on the ImageNet dataset, was chosen^16^. This architecture has shown excellent performance in imaging tasks, is readily available, and is medium-sized, which should fit the amount of data available well. Two different loss functions were tested for optimization: cross-entropy loss and focal loss (with parameters α = 1 and γ = 2). The latter was used because it should give more importance to examples that are harder to classify than cross-entropy loss^17^. The loss function was optimized using the Adam optimizer (with parameters β_1_ = 0.9, β_2_ = 0.999). Multiple augmentations like brightness changes, sharpening etc. were applied during training to synthetically increase the sample size, which helps the network to generalize better (A full list can be found in Supplemental 1). The batch size was set to 32. Training was stopped after 30 epochs. For development, PyTorch v1.4^18^ and PyTorch-lightning v1.2.6^19^ were used. Details on the network can be found in Supplemental 1.

### Cross-validation

One of the most important parameters when learning a neural network is the learning rate. Therefore, a fivefold cross-validation was employed to choose an optimal learning rate and the loss function. The accuracy of the test fold was used to measure the performance of the model. The learning rate that showed the highest accuracy was selected for training the final model.

### Validation

The final model was then retrained on the whole training data set with the corresponding learning rate and loss. The same training parameters were used, i.e., the training was performed with the Adam optimizer for 30 epochs with a batch size of 32. The performance on the final model was then measured on the internal and also on the external validation cohort.

To understand how the network derived its decisions from the radiographs, occlusion sensitivity maps^20^ with a stride of 8 and a patch size of 48 were employed to produce heat maps of the regions that contributed to a given decision.

### Statistics

All descriptive statistics were reported as mean +/− standard deviation or standard error where appropriate. Statistical significance was chosen to be below a *p*-value of 0.05. All analyses were conducted with Python 3.7 and the SciPy 1.5.4 package^21^.

## Results

### Patient collective

**Figure 3:**
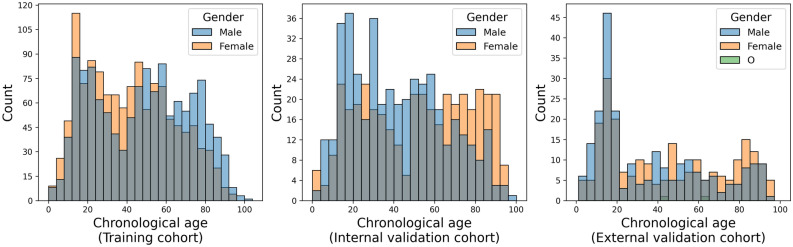
Histogram of the gender of the patient cohorts.

The mean age of all patients was 44.5 ± 24.4 years (range 0–101 years), with 1908 females and 1910 males (Table 2). There was not much difference in gender between patients of the training and validation cohorts, but there was a significant difference in age, especially for the external validation cohort (Fig. 3).

**Table 2:** Demographics of the patient collective.

	Training cohort [%]	Internal validation cohort [%]	External validation [%]	All [%]
Gender [F]	51% (1285/2540)	48% (391/816) (*P* = 0.20)	50% (232/462) (*P* = 0.86)	50% (1908/3818)
Age	44.5 +/−23.4 (range 0–101)	46.5 +/−24.9 (range 0–97) (*P* = 0.048)	40.7 +/−28.1 (range 1–97) (*P* < 0.001)	44.5 +/−24.4 (range 0–101)

The *P*-value denotes the significance of a chi-square and a Wilcoxon rank-sum test for sex and age between the training and the internal and external validation cohorts, respectively.

### Annotations

All knees in the radiographs were labeled to be either left and right. In the training cohort, 1403 left (48.5%) and 1489 right knees (51.5%) could be found, while in the internal validation there were 457 right (49%) and 475 left knees (51%). Similarly, in the external validation cohort, there were 260 left (57%) and 229 (43%) right knees. No significant difference between the training set and each validation cohort could be seen using chi-square tests (both *p* > 0.05).

While annotating, 11 radiographs of the training cohort were found to be mislabeled, i.e., the radiographer placed the wrong ASM on the radiograph, corresponding to 0.38% (11/2892) of all radiographs. In addition, in 1.3% (37/2892) no ASM could be found. Similarly, in the internal validation cohort in 2 cases a mislabeling was found (0.2%; 2/932) and there were 29 radiographs with no ASM, corresponding to 3.1% (29/932). In the external validation cohort, there was only 1 radiograph mislabeled (0.2%; 1/489), while 12 were missing an ASM (2.5%; 12/489).

### Cross-validation

**Figure 4:**
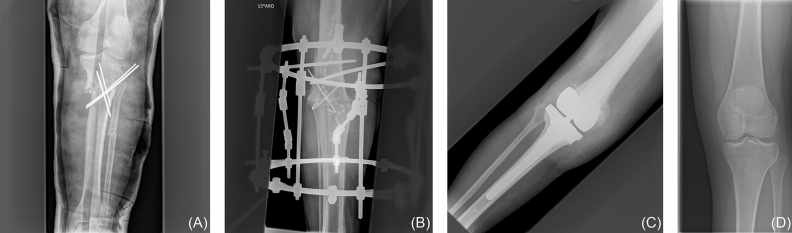
Four (of eight) cases that were misclassified during internal cross-validation by the best model using a learning rate of 3 × 10^–4^ and cross-entropy loss (**A**) male patient, 16 years (**B**) male patient, 81 years (**C**) female patient, 64 years (**D**) female patient, 15 years. The misclassifications of the network in (**A**)-(**C**) could be explained as being more complex cases. However, the misclassification in (**D**) seems not readily explainable.

The best learning rate was 3 × 10^–4^ using cross-entropy as a loss, with a mean accuracy of 99.7% (SE: 0.001%) (Table 3). On the 2892 images in the training set, the models trained with this learning rate made 8 errors altogether, 6 times a right knee was predicted to be a left knee, and 2 times vice versa (Fig. 4). The model was thus retrained on all training data with a learning rate of 3 × 10^–4^, cross-entropy loss and the same parameters otherwise.

**Table 3:** Mean accuracy and standard error of the models trained during cross-validation.

Learning rate	Model accuracy	
	Cross-entropy loss	Focal loss
9 × 10^–4^	99.5 +/−0.001	99.2 +/−0.002
6 × 10^–4^	99.5 +/−0.001	99.3 +/−0.001
3 × 10^–4^	**99.7 +/−0.001**	**99.6 +/−0.002**
1 × 10^–4^	99.6 +/−0.002	99.1 +/−0.002
9 × 10^–5^	99.6 +/−0.002	99.1 +/−0.001

The highest accuracy is marked in bold.

### Internal and external validation

**Figure 5:**
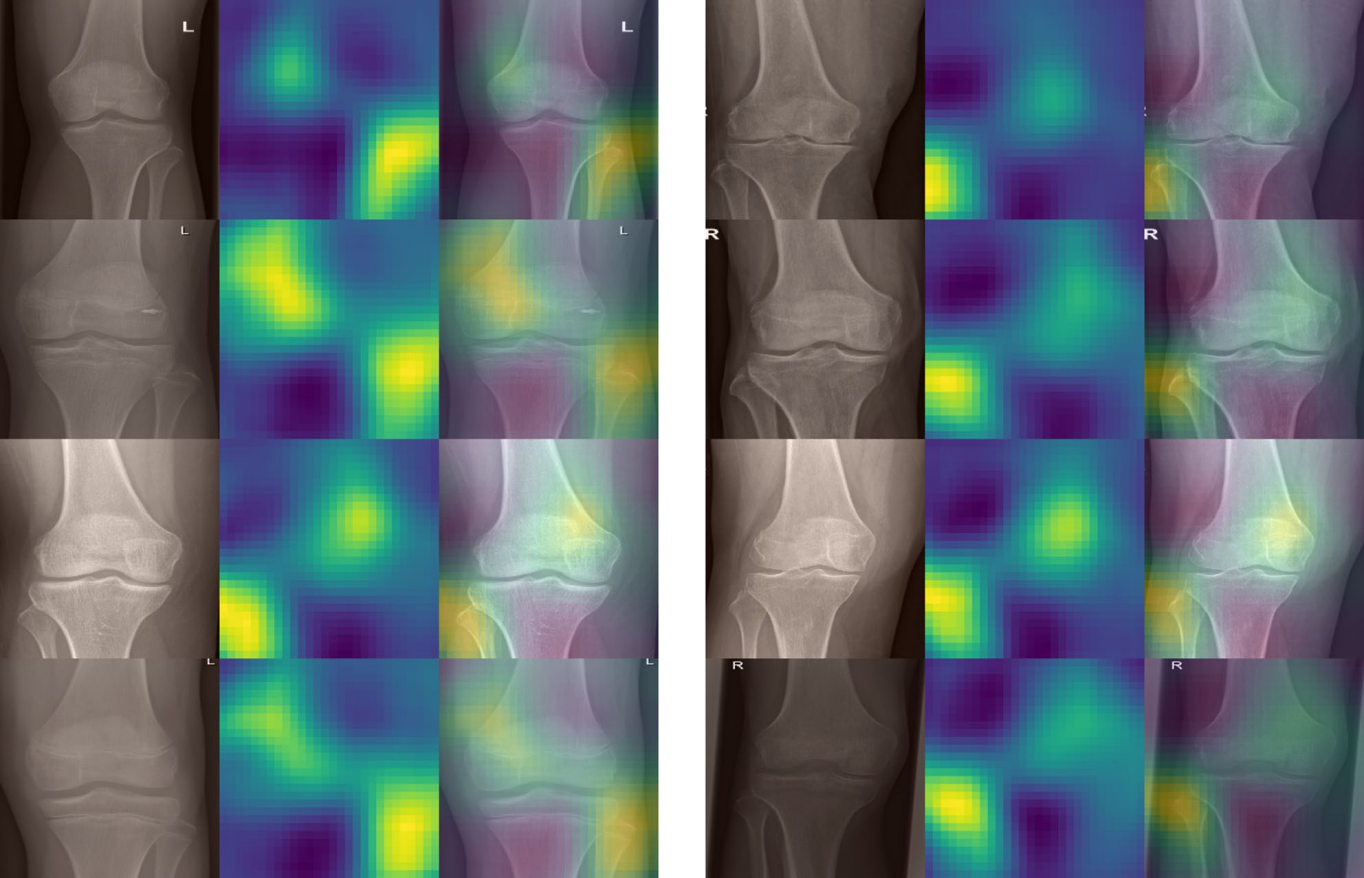
Occlusion sensitivity maps for randomly selected images from the internal validation cohort (left column) and external validation cohort (right column). In each row first the cropped, original image is shown, then the occlusion sensitivity map and finally an overlay of both. In general, two hot spots are visible, which correspond to the region that is most important for the network for its decision: the fibula as well as the lower end of the femur opposite to the fibula.

The final model did not misclassify any image on both the internal and external validation cohorts, thus showing perfect accuracy on all the 932 and 489 images. For 4 randomly selected images from each validation cohort, occlusion sensitivity maps were applied (Fig. 5).

## Discussion

Side marking in X-ray images is of great clinical importance to avoid side confusion with potentially fatal consequences. Whereas in the past a radiopaque side marker was placed in the examination area and also x-rayed, nowadays the side marking is increasingly done digitally. Until now, this is done manually by radiographers and is tedious work due to the sheer mass of radiographs. Due to the fact that the digital detectors used today cannot be exposed from the wrong side, unlike X-ray films or storage film systems in the past, it is possible to automate this task at least for skeletal images.

In our study we demonstrated that this manual work can equally well be performed by a neural network. We trained a standard network architecture, the ResNet-34, to determine the anatomical side in radiographs of the knee.

The network demonstrated excellent performance on an internal as well as external validation cohort. The accuracy on both cohort was slightly higher than during cross-validation, which might be due to chance. Nonetheless, the network seemed to be able to generalize to unseen data without compromising the overall accuracy.

When comparing the accuracy to those of the radiographers, the network performed similarly during the internal cross-validation. The radiographers had a mislabeling in 0.38% of all cases, which is nearly the same as the error of the network, which was 0.28% (*p* = 0.65, using a chi-square test). On the internal and external validation sets, the radiographers mislabeled in 0.2% of all cases. However, although the network did not show any error here, there is no statistically significant difference between them (*p* = 0.48 and *p* = 1.0). It, therefore cannot be claimed that the network performs better just because it showed perfect accuracy. In contrast to the radiographers, who missed placing the anatomical side markers in around 1.3%, 3.1%, and 2.5% of all cases, the network will always predict by design.

We applied the occlusion sensitivity maps for a rough estimation of which parts of the knee radiograph are important for the network to draw its estimation from. As expected, the fibula seems to be the most important part to determine the anatomical side of the radiograph. Nonetheless, unexpectedly in many images a second hot spot is visible, on the opposite side of the fibula and above the intercondylar area. The network seems to take into consideration the slant of the lower end of the femur, which seems also to be a rather good indication whether the knee is a left or right one. Despite this, we stress that any network is in general a black box and interpretation has to be taken with some care.

Although anatomical side and placing ASMs is a routine task, there are only very few studies that automatically try to predict anatomical side in radiographs: For chest radiographers, Xue et al. present a system based on classical image processing to predict if a radiograph shows the chest in frontal or lateral view^22^, while Reza et al. predict the projection (PA vs AP), also using more classical image processing and machine learning techniques^23^. Fang et al. present a more general network that classifies the view in general radiographs^24^. Unfortunately, as the problem is much wider, naturally their accuracy is lower. They also removed radiographs of children, which often have much higher variability. Other studies concentrate on the radiological findings, for example, several studies present a deep network for generating labels that can be directly used in radiological reports^25,26^. These models assume the correct anatomical side, which could be checked using a network like the one we have presented.

Determining the anatomical side of the knee in AP views seems a rather easy task as long as the fibula is visible. Despite this, biological variability and the acquisition conditions are very different, as scans may contain screws, implants, cast and other temporary stabilization artifacts. This variation makes an automation using classical image processing and machine learning techniques rather complex and error prone. Neural networks on the other hand are much better suited for the task and our results show that also an off-the-shelf network can solve such problems easily.

We restricted ourselves to knee radiographs acquired in AP direction, since this is the most common acquisition in clinical routine, and our network shows perfect accuracy in two validation cohorts, equaling those of radiographers and making application in clinical routine possible. However, because a wrong ASM can have severe consequences and deep learning networks are in general black boxes, the prediction of the network could be presented to the radiographer for a second check, making mistakes even less probable.

We have little doubt that similar networks to determine the anatomical side for other body parts such as chest, abdomen, spine, upper and lower extremities etc. as well as in lateral plan or AP view and lateral view combined can very easily be developed with similarly high accuracies. Such studies should be performed in future. Since the internal and external validation cohorts were acquired with different scanners but represent a similar population, it should be also verified that the network performs equally well in other populations.

Our cohorts were acquired from the clinical routine and were only randomly subsampled to reduce the sample size. Despite this, a statistically significant age difference was observed. While we have no clear explanation for this difference, since the population of the hospitals should be relatively similar, this is not a disadvantage to our study. Indeed, our network still performed excellent, showing that it can work with images of patients of any age.

In our experiments, we used the ResNet-34 network because it is a medium-sized network and has performed excellently in many applications. However, we believe that other networks, e.g., the Inception V3 or the VGG-16 network, would work equally well. Similarly, contrary to our expectation, the cross-entropy loss performed better than the focal loss in our experiment. It might be because the focal loss has two parameters, which we could have selected suboptimally and which we did not tune during cross-validation. Nonetheless, although the cross-entropy loss was better during cross-validation, showing only 8 instead of 14 errors, this difference is not statistically significant (*p* = 0.29, using a chi-square test).

Some limitations apply to our study: Even though we used radiographs from two validation cohorts obtained from scanners from different vendors, it is necessary to check that the network will perform equally well on different populations. In addition, while the restriction to AP views, while by far the most often used view direction in clinical routine, a network that can deal with PA views as well would be desirable. In addition, we assumed that the radiographs were acquired with a computed radiography system and therefore no mirroring occurred when the images were transferred to the PACS, which is more common when using film-screen radiography.

In conclusion, a neural network to determine the anatomical side in radiographs of the knee was trained and was shown to perform excellently in two validation cohorts.

## Supplementary Information

Supplementary Information.

## Data Availability

The datasets generated during and/or analyzed during the current study are available from the corresponding author upon reasonable request.
