# Supplementary material for: Determining the anatomical site in knee radiographs using deep learning
Source: Sci Rep. 2022 Mar 7;12:3995. doi: 10.1038/s41598-022-08020-7 (PMC8900105; doi:10.1038/s41598-022-08020-7)
Supplement: Supplementary file 1 — Supplementary Information. [file 41598_2022_8020_MOESM1_ESM.docx]

Supplemental Digital Content 1

For modeling, the ResNet-34, pretrained on ImageNet, was used as it has shown excellent performance in several tasks and is easily available in multiple frameworks. Since the pretrained network has 1000 output nodes, two fully connected layers were used with an ReLU activation function in between. The first layer reduced the number from 1000 nodes to 250, while the second reduced it further to two output nodes. As a loss functions, cross entropy and Focal Loss was employed. Focal loss is supposed to have the advantage that it resembles “hard negative mining”, which could be important in our case as apart from the normal knee, a few knees had implants and casts. For the focal loss, the parameters α = 1 and γ = 2 were chosen. Optimization was done with the Adam optimizer with default parameters (beta1 = 0.9, beta2 = 0.999, eps = 1e-08, weight_decay = 0, amsgrad = False). The batch size was fixed to 32 and training was performed for 30 epochs. Gradients of 5 batches were accumulated during training to increase stability, in addition, Stochastic Weight Averaging was employed (number of annealing epochs of 10, with cosine annealing strategy).

Radiographs were first randomly cropped to a size of 224x224 before training. Several augmentations were then applied: With 50% chance a Coarse Dropout (i.e. putting a black rectangle into the area) with maximum width and height of 1/10^th^ of the image, a CLAHE transform with clipLimit of 4.0 and grid size of 8x8, a random gamma transformation with limits between 70 and 130, a random brightness contrast with brightness and contrast limits of 0.2, a sharpening filter with alpha between 0.2 and 0.5 and lightness between 0.5 and 1.0 as well as a blur filter with blur limit of 7 was applied. Then an elastic transform with no affine component and a rotation with maximum degree of 22 and with probability of 0.2 was applied. No random flip was applied, as this would change the laterality in AP images. Then the images were intensity normalized to the range -1,1.

For predicting, the images were similarly processed, but no augmentations were used: First they were resized to a size of 256x256 and then center cropped to a size of 224x224. After this, the intensity was normalized before the image was processed by the network.

To develop the network, Python 3.7, PyTorch 1.4.0, PyTorch Lightning 1.2.6 as well as the OpenCV and Albumentation frameworks were utilized. The torchvision library was used to obtain the pretrained weights for the network. Visualization of the occlusion sensitivity maps were built using the Captum framework. Commodity hardware was used for training (AMD Ryzen Threadripper 2950X with 128GB of RAM, a 1 TB SSD drive and an NVidia TITAN RTX card, running Ubuntu 18.04 LTS). The source code of the experiments will be published in a repository on github [https://github.com/aydindemircioglu/knee.laterality].
